# *cis*-(1,4,8,11-Tetra­aza­cyclo­tetra­decane-κ*N* ^4^)bis(­thio­cyanato-κ*N*)chromium(III) thio­cyanate

**DOI:** 10.1107/S1600536813015456

**Published:** 2013-06-12

**Authors:** Dohyun Moon, Jong-Ha Choi, Keon Sang Ryoo, Yong Pyo Hong

**Affiliations:** a Pohang Accelerator Laboratory, POSTECH, Pohang 790-784, Republic of Korea; b Department of Chemistry, Andong National University, Andong 760-749, Republic of Korea

## Abstract

The crystal structure of [Cr(NCS)_2_(cyclam)]NCS (cyclam = 1,4,8,11-tetra­aza­cyclo­tetra­deca­ne, C_10_H_24_N_4_) has been determined by using synchrotron radiation at 98 K. The Cr^III^ atom is in a slightly distorted octa­hedral environment with four N atoms of the macrocyclic ligand and two N-coordinated NCS^−^ anions in *cis* positions. The average Cr—N(cyclam) and Cr—NCS bond lengths are 2.085 (5) and 1.996 (15) Å, respectively. In the crystal, the uncoordinating SCN^−^ anion is hydrogen bonded through N—H⋯S and N—H⋯N inter­actions to neighbouring complex cations.

## Related literature

For the synthesis, see: Ferguson & Tobe (1970 ▶); For spectroscopic studies, see: Choi & Park (2003 ▶); Poon & Pun (1980 ▶). For related structures, see: Forsellini *et al.* (1986 ▶); Friesen *et al.* (1997 ▶); Meyer *et al.* (1998 ▶); Choi *et al.* (2004*a* ▶,*b* ▶, 2009 ▶); Subhan *et al.* (2011 ▶).
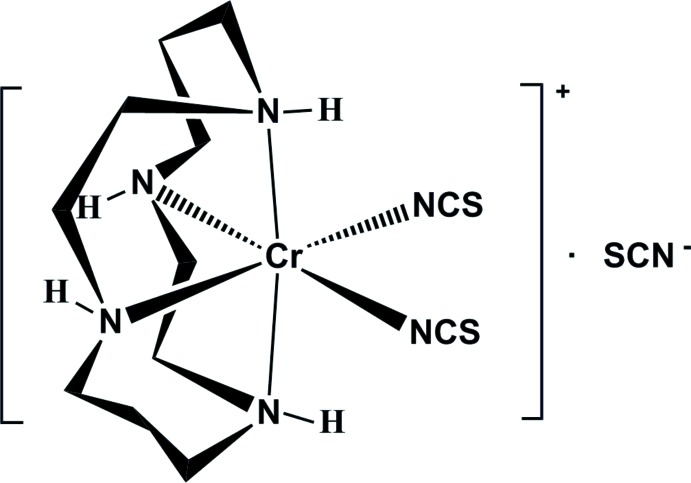


## Experimental

### 

#### Crystal data

[Cr(NCS)_2_(C_10_H_24_N_4_)]NCS
*M* *_r_* = 426.57
Monoclinic, 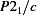

*a* = 10.590 (2) Å
*b* = 7.6970 (15) Å
*c* = 23.750 (5) Å
β = 94.70 (3)°
*V* = 1929.4 (7) Å^3^
*Z* = 4
Synchrotron radiation
λ = 0.740 Å
μ = 1.03 mm^−1^
*T* = 98 K
0.01 × 0.01 × 0.01 mm

#### Data collection

ADSC Q210 CCD area-detector diffractometer
Absorption correction: empirical (*HKL-3000* *SCALEPACK*; Otwinowski & Minor, 1997 ▶) *T*_min_ = 0.988, *T*_max_ = 0.989
16587 measured reflections
4727 independent reflections
3998 reflections with *I* > 2σ(*I*)
*R*_int_ = 0.038

#### Refinement

*R*[*F* ^2^ > 2σ(*F* ^2^)] = 0.030
*wR*(*F* ^2^) = 0.085
*S* = 1.07
4727 reflections
234 parameters
H atoms treated by a mixture of independent and constrained refinement
Δρ_max_ = 0.38 e Å^−3^
Δρ_min_ = −0.46 e Å^−3^

### 

Data collection: *ADSC Quantum-210 ADX* (Arvai & Nielsen, 1983 ▶); cell refinement: *HKL-3000* (Otwinowski & Minor, 1997 ▶); data reduction: *HKL-3000*; program(s) used to solve structure: *SHELXS2013* (Sheldrick, 2008) ▶; program(s) used to refine structure: *SHELXL2013* (Sheldrick, 2008) ▶; molecular graphics: *DIAMOND* (Brandenburg, 2007 ▶); software used to prepare material for publication: *WinGX* (Farrugia, 2012 ▶).

## Supplementary Material

Crystal structure: contains datablock(s) I. DOI: 10.1107/S1600536813015456/lr2107sup1.cif

Structure factors: contains datablock(s) I. DOI: 10.1107/S1600536813015456/lr2107Isup2.hkl

Additional supplementary materials:  crystallographic information; 3D view; checkCIF report

## Figures and Tables

**Table 1:** Hydrogen-bond geometry (Å, °)

*D*—H⋯*A*	*D*—H	H⋯*A*	*D*⋯*A*	*D*—H⋯*A*
N1—H1*N*1⋯S2^i^	0.86 (2)	2.59 (2)	3.4138 (15)	160.1 (17)
N2—H1*N*2⋯S4^ii^	0.77 (2)	2.66 (2)	3.3521 (14)	149.8 (18)
N3—H1*N*3⋯N7^ii^	0.834 (19)	2.119 (19)	2.9238 (19)	162.0 (17)
N4—H1*N*4⋯N7^iii^	0.851 (19)	2.150 (19)	2.947 (2)	155.9 (17)

Symmetry codes: (i) 
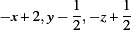
; (ii) 
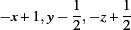
; (iii) 
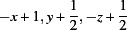
.

**Fig. 1.**
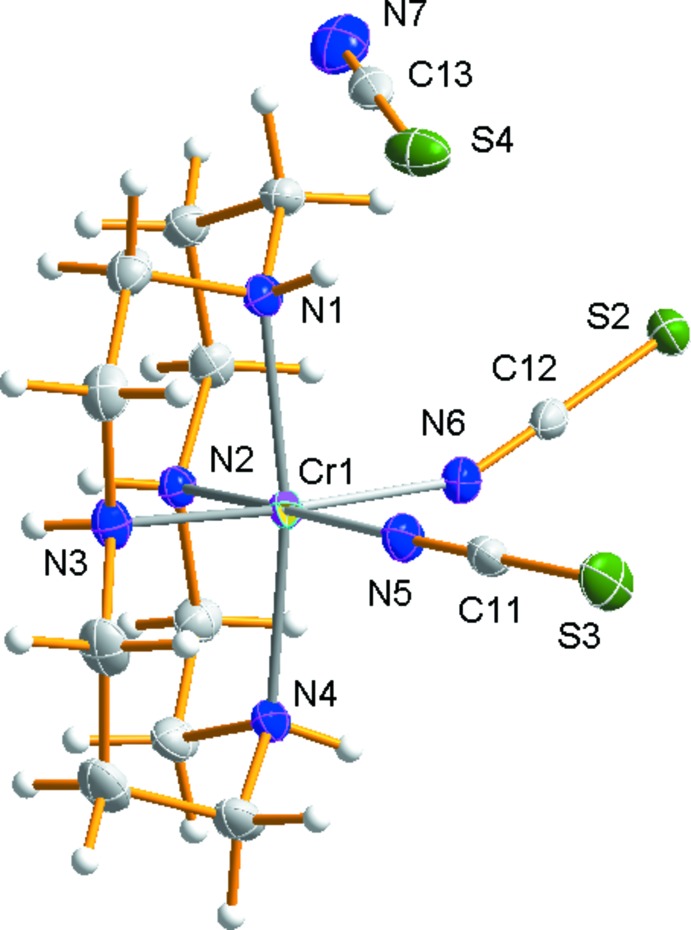
Perspective view (50% probablility level) of cis-[Cr(cyclam)(NCS)2]SCN

## supplementary crystallographic information

### Comment

The cyclam (1,4,8,11-tetraazacyclotetradecane) ligand is moderately flexible structure, and can adopt both planer (*trans*) and folded (*cis*) configurations (Poon & Pun, 1980). There are five conformational *trans* isomers for the cyclam which differ in the chirality of the *sec*-NH centers. The *trans*-I, *trans*-II and *trans*-V configurations can fold to form *cis*-I, *cis*-II and *cis*-V isomers, respectively (Subhan *et al.*, 2011). Furthermore, the NCS group is an ambidentate ligand because it can coordinate to a transition metal ion through the nitrogen (*M*-NCS), or the sulfur (*M*-SCN), or both (*M*-NCS-*M*).

In this communication, we report the structure of [Cr(cyclam)(NCS)_2_]SCN in order to determine the mode of bonding of the thiocyanate group and to verify geometrical assignment made on the basis of spectroscopic measurements (Poon & Pun, 1980; Choi & Park (2003).

Counter anionic species play a very important role in coordination chemistry. This is another example of a *cis*-[Cr(cyclam)_2_(NCS)_2_]^+^ but with different counter anion (Friesen *et al.*, 1997). The structural analysis shows that there is only one crystallographically independent Cr(III) complex cation where the nitrogen atoms of cyclam ligand occupy four adjacent sites and the two N-bonded NCS groups coordinate to the chromium centre in *cis* arrangement. The cyclam adopts the folded *cis*-V configuration with six- and five-membered chelate rings in *chair* and *gauche* conformation, respectively*.* The same conformational arrangement has been found in *cis*-[Cr(cyclam)(ONO)_2_]NO_2_(Choi *et al.*, 2004*a*). An ellipsoid plot (50% probability level) of the *cis*-[Cr(cyclam)(NCS)_2_]SCN, together with the atomic labelling, is depicted in Fig. 1.

The Cr—N(cyclam) distances of 2.0851 (14) and 2.0897 (14) Å are good agreement with the corresponding Cr—N distances found in [Cr(cyclam)(ox)]ClO_4_ (Choi *et al.*, 2004*b*), [Cr(cyclam)(acac)](ClO_4_)_2_ (Subhan *et al.* , 2011) and *trans*-[Cr(cyclam)(nic-*O*)_2_]ClO_4_ (Choi, 2009). The mean Cr-NCS distance of 1.9957 (14) Å is close the value of the range 1.9827 (15)–1.9895 (16) Å found in *trans*-[Cr(Me_2_tn)_2_(NCS)_2_]SCN, but slightly longer than the 1.9698 (14) Å of Cr-ONO found in *cis*-[Cr(cyclam)(ONO)_2_]NO_2_ (Choi *et al.*, 2004*a*). The folded angle of 97.11° in the cyclam is comparable to the corresponding angles of 98.55°, 97.03°, 95.09°, 94.51° and 92.8° in [Cr(cyclam)(ox)]ClO_4_, [Cr(cyclam)(acac)](ClO_4_)_2_, *cis*-[Cr(cyclam)(ONO)_2_]NO_2_, *cis-*[Cr(cyclam)(N_3_)_2_]ClO_4_ and *cis-*[Cr(cyclam)Cl_2_]Cl, respectively (Choi *et al.*, 2004*b*; Subhan *et al.*, 2011; Meyer *et al.*, 1998; Forsellini *et al.*, 1986). As usually observed, the five-membered chelate rings adopt a *gauche*, and six-membered ring is in the chair conformation. The average bond angles of five- and six-membered chelate rings around chromium(III) are the 83.13 (6) and 90.21 (6)°, respectively. The coordinated isothiocyanate ligands are almost linear with N—C—S angles of 179.69 (17)° and 178.83 (15)°. The uncoordinated NCS^-^ anion also adopts a linear conformation and its N and S atoms participates in hydrogen bonds with the N—H groups of cyclam ligand. The C12—S2 and C13—S4 bond lengths [1.6232 (17) and 1.639 (2) Å] of are slightly shorter than the C11—S3 [1.6082 (17) Å] in the NCS^-^ groups. It seems that the slight elongation of the distances are attributed to the weak H atoms bonds of both S2 and S3 atoms. Table 1 contains the distances and angles of hydrogen bonds. These hydrogen-bonded networks help to stabilize the crystal structure.

### Experimental

The free ligand cyclam was purchased from Stream Chemicals and used as provided. All chemicals were reagent grade materials and used without further purification. The *cis*-[Cr(cyclam)(NCS)_2_]SCN was synthesized according to the literature (Ferguson & Tobe, 1970).

### Refinement

Non-hydrogen atoms were refined anisotropically; hydrogen atoms were first located in a difference map; N–H hydrogen atoms were freely refined and C–H hydrogen atoms were constrained to ride on the parent carbon atom, with C–H = 0.98 Å and C–H = 0.99 Å and *U*_iso_(H) = 1.2*U*_eq_(C) for methylene groups.

### Crystal data

**Table:**

[Cr(NCS)_2_(C_10_H_24_N_4_)]NCS	*F*(000) = 89
*M**_r_* = 426.57	*D*_x_ = 1.469 Mg m^−^^3^
Monoclinic, *P*2_1_/*c*	Synchrotron radiation, λ = 0.740 Å
*a* = 10.590 (2) Å	Cell parameters from 39021 reflections
*b* = 7.6970 (15) Å	θ = 1.9–33.1°
*c* = 23.750 (5) Å	µ = 1.03 mm^−^^1^
β = 94.70 (3)°	*T* = 98 K
*V* = 1929.4 (7) Å^3^	Block, pink
*Z* = 4	0.01 × 0.01 × 0.01 mm

### Data collection

**Table:**

ADSC Q210 CCD area-detector diffractometer	3998 reflections with *I* > 2σ(*I*)
Radiation source: PLSII 2D bending magnet	*R*_int_ = 0.038
ω scan	θ_max_ = 29.5°, θ_min_ = 2.0°
Absorption correction: empirical (using intensity measurements) (*HKL-3000**SCALEPACK*; Otwinowski & Minor, 1997)	*h* = −14→14
*T*_min_ = 0.988, *T*_max_ = 0.989	*k* = −10→10
16587 measured reflections	*l* = −31→31
4727 independent reflections	

### Refinement

**Table:**

Refinement on *F*^2^	Hydrogen site location: inferred from neighbouring sites
Least-squares matrix: full	H atoms treated by a mixture of independent and constrained refinement
*R*[*F*^2^ > 2σ(*F*^2^)] = 0.030	*w* = 1/[σ^2^(*F*_o_^2^) + (0.0543*P*)^2^ + 0.0369*P*] where *P* = (*F*_o_^2^ + 2*F*_c_^2^)/3
*wR*(*F*^2^) = 0.085	(Δ/σ)_max_ = 0.001
*S* = 1.07	Δρ_max_ = 0.38 e Å^−^^3^
4727 reflections	Δρ_min_ = −0.46 e Å^−^^3^
234 parameters	Extinction correction: *SHELXL*, Fc^*^=kFc[1+0.001xFc^2^λ^3^/sin(2θ)]^-1/4^
0 restraints	Extinction coefficient: 0.0149 (11)

### Special details

**Table:**

Geometry. All e.s.d.'s (except the e.s.d. in the dihedral angle between two l.s. planes) are estimated using the full covariance matrix. The cell e.s.d.'s are taken into account individually in the estimation of e.s.d.'s in distances, angles and torsion angles; correlations between e.s.d.'s in cell parameters are only used when they are defined by crystal symmetry. An approximate (isotropic) treatment of cell e.s.d.'s is used for estimating e.s.d.'s involving l.s. planes.

### Fractional atomic coordinates and isotropic or equivalent isotropic displacement parameters (Å^2^)

**Table:**

	*x*	*y*	*z*	*U*_iso_*/*U*_eq_	
Cr1	0.78572 (2)	0.65230 (3)	0.13425 (2)	0.01445 (9)	
S2	0.80594 (3)	1.01716 (5)	0.29372 (2)	0.02043 (10)	
S3	1.17137 (4)	0.91514 (6)	0.09132 (2)	0.03337 (12)	
N1	0.87142 (12)	0.48585 (16)	0.19523 (5)	0.0176 (3)	
H1N1	0.949 (2)	0.521 (2)	0.2008 (8)	0.030 (5)*	
N2	0.60770 (11)	0.58345 (16)	0.15912 (5)	0.0158 (2)	
H1N2	0.5895 (18)	0.494 (3)	0.1465 (8)	0.026 (5)*	
N3	0.80817 (12)	0.44123 (17)	0.08083 (5)	0.0194 (3)	
H1N3	0.7441 (18)	0.378 (2)	0.0769 (8)	0.024 (5)*	
N4	0.67930 (12)	0.78077 (18)	0.06965 (5)	0.0193 (3)	
H1N4	0.6773 (17)	0.888 (2)	0.0783 (8)	0.024 (5)*	
N5	0.95059 (12)	0.74035 (17)	0.11200 (5)	0.0216 (3)	
N6	0.78208 (12)	0.85015 (16)	0.18914 (5)	0.0203 (3)	
C1	0.82134 (14)	0.4809 (2)	0.25209 (6)	0.0199 (3)	
H1A	0.8357	0.5954	0.2705	0.024*	
H1B	0.8694	0.3932	0.2756	0.024*	
C2	0.68044 (14)	0.4373 (2)	0.25004 (6)	0.0203 (3)	
H2A	0.6664	0.3245	0.2305	0.024*	
H2B	0.6569	0.4225	0.2892	0.024*	
C3	0.59240 (14)	0.5705 (2)	0.22082 (6)	0.0195 (3)	
H3A	0.5037	0.5387	0.2263	0.023*	
H3B	0.6094	0.6855	0.2385	0.023*	
C4	0.88174 (15)	0.30950 (19)	0.17019 (7)	0.0224 (3)	
H4A	0.8016	0.2449	0.1728	0.027*	
H4B	0.9509	0.2437	0.1911	0.027*	
C5	0.90900 (15)	0.3290 (2)	0.10901 (7)	0.0248 (3)	
H5A	0.9932	0.3830	0.1064	0.030*	
H5B	0.9089	0.2138	0.0905	0.030*	
C6	0.51544 (14)	0.7103 (2)	0.13191 (6)	0.0215 (3)	
H6A	0.5239	0.8239	0.1514	0.026*	
H6B	0.4278	0.6677	0.1341	0.026*	
C7	0.54392 (14)	0.7294 (2)	0.07095 (6)	0.0228 (3)	
H7A	0.5283	0.6180	0.0507	0.027*	
H7B	0.4884	0.8191	0.0521	0.027*	
C8	0.83850 (16)	0.4808 (2)	0.02215 (6)	0.0266 (3)	
H8A	0.8490	0.3703	0.0017	0.032*	
H8B	0.9201	0.5441	0.0235	0.032*	
C9	0.73707 (17)	0.5890 (2)	−0.01027 (7)	0.0295 (4)	
H9A	0.7568	0.5952	−0.0503	0.035*	
H9B	0.6548	0.5286	−0.0093	0.035*	
C10	0.72283 (16)	0.7724 (2)	0.01150 (6)	0.0257 (3)	
H10A	0.8054	0.8326	0.0114	0.031*	
H10B	0.6614	0.8356	−0.0147	0.031*	
C11	1.04332 (14)	0.81402 (19)	0.10314 (6)	0.0200 (3)	
C12	0.79292 (13)	0.92117 (18)	0.23267 (6)	0.0172 (3)	
S4	0.56857 (4)	0.72414 (6)	0.36233 (2)	0.03051 (12)	
N7	0.37447 (15)	0.65647 (19)	0.43301 (7)	0.0338 (3)	
C13	0.45576 (15)	0.68218 (19)	0.40360 (7)	0.0235 (3)	

### Atomic displacement parameters (Å^2^)

**Table:**

	*U*^11^	*U*^22^	*U*^33^	*U*^12^	*U*^13^	*U*^23^
Cr1	0.01319 (12)	0.01531 (13)	0.01455 (13)	−0.00352 (8)	−0.00060 (8)	0.00023 (8)
S2	0.01916 (18)	0.02155 (19)	0.02016 (19)	−0.00004 (13)	−0.00094 (14)	−0.00355 (14)
S3	0.0250 (2)	0.0448 (3)	0.0303 (2)	−0.01871 (19)	0.00255 (17)	0.00597 (19)
N1	0.0142 (6)	0.0178 (6)	0.0205 (6)	−0.0020 (5)	−0.0012 (5)	0.0014 (5)
N2	0.0142 (6)	0.0153 (6)	0.0176 (6)	−0.0019 (5)	0.0005 (5)	−0.0001 (5)
N3	0.0170 (6)	0.0210 (6)	0.0205 (6)	−0.0036 (5)	0.0031 (5)	−0.0031 (5)
N4	0.0183 (6)	0.0204 (6)	0.0184 (6)	−0.0053 (5)	−0.0028 (5)	0.0018 (5)
N5	0.0183 (6)	0.0244 (7)	0.0218 (6)	−0.0068 (5)	0.0002 (5)	0.0017 (5)
N6	0.0216 (6)	0.0171 (6)	0.0217 (6)	−0.0036 (5)	−0.0012 (5)	−0.0002 (5)
C1	0.0212 (7)	0.0209 (7)	0.0171 (7)	−0.0012 (6)	−0.0019 (6)	0.0035 (6)
C2	0.0213 (7)	0.0213 (7)	0.0183 (7)	−0.0019 (6)	0.0022 (6)	0.0036 (6)
C3	0.0176 (7)	0.0223 (7)	0.0190 (7)	−0.0020 (6)	0.0045 (6)	−0.0001 (6)
C4	0.0208 (7)	0.0177 (7)	0.0282 (8)	0.0016 (6)	0.0000 (6)	0.0006 (6)
C5	0.0219 (7)	0.0231 (8)	0.0298 (8)	0.0023 (6)	0.0042 (6)	−0.0043 (6)
C6	0.0154 (7)	0.0230 (7)	0.0256 (8)	0.0005 (6)	−0.0006 (6)	0.0038 (6)
C7	0.0172 (7)	0.0275 (8)	0.0228 (7)	−0.0041 (6)	−0.0045 (6)	0.0043 (6)
C8	0.0293 (8)	0.0320 (9)	0.0195 (7)	−0.0042 (7)	0.0077 (6)	−0.0062 (6)
C9	0.0340 (9)	0.0378 (9)	0.0167 (7)	−0.0073 (8)	0.0013 (7)	−0.0040 (7)
C10	0.0284 (8)	0.0323 (9)	0.0160 (7)	−0.0071 (7)	−0.0006 (6)	0.0049 (6)
C11	0.0211 (7)	0.0234 (7)	0.0150 (7)	−0.0028 (6)	−0.0019 (6)	0.0004 (6)
C12	0.0146 (6)	0.0146 (6)	0.0218 (7)	−0.0024 (5)	−0.0017 (6)	0.0028 (5)
S4	0.0347 (2)	0.0333 (2)	0.0235 (2)	0.01233 (18)	0.00211 (17)	0.00132 (17)
N7	0.0297 (8)	0.0263 (7)	0.0453 (9)	0.0070 (6)	0.0013 (7)	0.0027 (6)
C13	0.0260 (8)	0.0160 (7)	0.0267 (8)	0.0081 (6)	−0.0087 (7)	−0.0027 (6)

### Geometric parameters (Å, º)

**Table:**

Cr1—N5	1.9846 (13)	C2—C3	1.515 (2)
Cr1—N6	2.0071 (13)	C2—H2A	0.9900
Cr1—N4	2.0781 (14)	C2—H2B	0.9900
Cr1—N1	2.0849 (13)	C3—H3A	0.9900
Cr1—N3	2.0868 (13)	C3—H3B	0.9900
Cr1—N2	2.0895 (13)	C4—C5	1.512 (2)
S2—C12	1.6231 (15)	C4—H4A	0.9900
S3—C11	1.6081 (16)	C4—H4B	0.9900
N1—C4	1.4895 (19)	C5—H5A	0.9900
N1—C1	1.4913 (19)	C5—H5B	0.9900
N1—H1N1	0.86 (2)	C6—C7	1.510 (2)
N2—C6	1.4904 (19)	C6—H6A	0.9900
N2—C3	1.4909 (18)	C6—H6B	0.9900
N2—H1N2	0.77 (2)	C7—H7A	0.9900
N3—C8	1.4871 (19)	C7—H7B	0.9900
N3—C5	1.489 (2)	C8—C9	1.518 (2)
N3—H1N3	0.834 (19)	C8—H8A	0.9900
N4—C7	1.4901 (19)	C8—H8B	0.9900
N4—C10	1.4928 (19)	C9—C10	1.515 (2)
N4—H1N4	0.851 (19)	C9—H9A	0.9900
N5—C11	1.168 (2)	C9—H9B	0.9900
N6—C12	1.1666 (19)	C10—H10A	0.9900
C1—C2	1.526 (2)	C10—H10B	0.9900
C1—H1A	0.9900	S4—C13	1.6384 (19)
C1—H1B	0.9900	N7—C13	1.169 (2)
			
N5—Cr1—N6	88.74 (6)	H2A—C2—H2B	107.5
N5—Cr1—N4	94.39 (5)	N2—C3—C2	112.52 (12)
N6—Cr1—N4	94.61 (6)	N2—C3—H3A	109.1
N5—Cr1—N1	93.01 (5)	C2—C3—H3A	109.1
N6—Cr1—N1	92.61 (5)	N2—C3—H3B	109.1
N4—Cr1—N1	169.77 (5)	C2—C3—H3B	109.1
N5—Cr1—N3	87.56 (6)	H3A—C3—H3B	107.8
N6—Cr1—N3	174.14 (5)	N1—C4—C5	108.61 (12)
N4—Cr1—N3	90.20 (5)	N1—C4—H4A	110.0
N1—Cr1—N3	83.06 (5)	C5—C4—H4A	110.0
N5—Cr1—N2	174.70 (5)	N1—C4—H4B	110.0
N6—Cr1—N2	86.73 (5)	C5—C4—H4B	110.0
N4—Cr1—N2	83.23 (5)	H4A—C4—H4B	108.3
N1—Cr1—N2	89.96 (5)	N3—C5—C4	107.65 (12)
N3—Cr1—N2	97.17 (5)	N3—C5—H5A	110.2
C4—N1—C1	112.42 (11)	C4—C5—H5A	110.2
C4—N1—Cr1	108.95 (9)	N3—C5—H5B	110.2
C1—N1—Cr1	118.54 (9)	C4—C5—H5B	110.2
C4—N1—H1N1	104.3 (13)	H5A—C5—H5B	108.5
C1—N1—H1N1	105.8 (13)	N2—C6—C7	107.70 (12)
Cr1—N1—H1N1	105.6 (13)	N2—C6—H6A	110.2
C6—N2—C3	110.46 (12)	C7—C6—H6A	110.2
C6—N2—Cr1	106.62 (9)	N2—C6—H6B	110.2
C3—N2—Cr1	117.91 (9)	C7—C6—H6B	110.2
C6—N2—H1N2	106.5 (14)	H6A—C6—H6B	108.5
C3—N2—H1N2	106.1 (14)	N4—C7—C6	108.29 (12)
Cr1—N2—H1N2	108.7 (14)	N4—C7—H7A	110.0
C8—N3—C5	109.76 (12)	C6—C7—H7A	110.0
C8—N3—Cr1	117.03 (10)	N4—C7—H7B	110.0
C5—N3—Cr1	106.96 (10)	C6—C7—H7B	110.0
C8—N3—H1N3	104.5 (13)	H7A—C7—H7B	108.4
C5—N3—H1N3	105.0 (12)	N3—C8—C9	112.93 (13)
Cr1—N3—H1N3	113.0 (13)	N3—C8—H8A	109.0
C7—N4—C10	112.23 (12)	C9—C8—H8A	109.0
C7—N4—Cr1	108.83 (9)	N3—C8—H8B	109.0
C10—N4—Cr1	118.20 (10)	C9—C8—H8B	109.0
C7—N4—H1N4	102.1 (12)	H8A—C8—H8B	107.8
C10—N4—H1N4	106.4 (12)	C10—C9—C8	115.10 (13)
Cr1—N4—H1N4	107.8 (12)	C10—C9—H9A	108.5
C11—N5—Cr1	170.02 (13)	C8—C9—H9A	108.5
C12—N6—Cr1	157.72 (12)	C10—C9—H9B	108.5
N1—C1—C2	113.34 (12)	C8—C9—H9B	108.5
N1—C1—H1A	108.9	H9A—C9—H9B	107.5
C2—C1—H1A	108.9	N4—C10—C9	113.77 (13)
N1—C1—H1B	108.9	N4—C10—H10A	108.8
C2—C1—H1B	108.9	C9—C10—H10A	108.8
H1A—C1—H1B	107.7	N4—C10—H10B	108.8
C3—C2—C1	115.42 (12)	C9—C10—H10B	108.8
C3—C2—H2A	108.4	H10A—C10—H10B	107.7
C1—C2—H2A	108.4	N5—C11—S3	179.66 (15)
C3—C2—H2B	108.4	N6—C12—S2	178.82 (14)
C1—C2—H2B	108.4	N7—C13—S4	178.35 (14)
			
C4—N1—C1—C2	−72.13 (15)	C3—N2—C6—C7	174.88 (12)
Cr1—N1—C1—C2	56.50 (15)	Cr1—N2—C6—C7	45.63 (13)
N1—C1—C2—C3	−64.97 (17)	C10—N4—C7—C6	169.85 (13)
C6—N2—C3—C2	177.56 (12)	Cr1—N4—C7—C6	37.10 (15)
Cr1—N2—C3—C2	−59.54 (15)	N2—C6—C7—N4	−55.65 (16)
C1—C2—C3—N2	66.51 (17)	C5—N3—C8—C9	177.66 (13)
C1—N1—C4—C5	169.43 (12)	Cr1—N3—C8—C9	−60.26 (16)
Cr1—N1—C4—C5	35.94 (14)	N3—C8—C9—C10	66.73 (19)
C8—N3—C5—C4	173.69 (12)	C7—N4—C10—C9	−71.42 (17)
Cr1—N3—C5—C4	45.79 (14)	Cr1—N4—C10—C9	56.52 (16)
N1—C4—C5—N3	−54.89 (16)	C8—C9—C10—N4	−64.47 (18)

### Hydrogen-bond geometry (Å, º)

**Table:**

*D*—H···*A*	*D*—H	H···*A*	*D*···*A*	*D*—H···*A*
N1—H1*N*1···S2^i^	0.86 (2)	2.59 (2)	3.4138 (15)	160.1 (17)
N2—H1*N*2···S4^ii^	0.77 (2)	2.66 (2)	3.3521 (14)	149.8 (18)
N3—H1*N*3···N7^ii^	0.834 (19)	2.119 (19)	2.9238 (19)	162.0 (17)
N4—H1*N*4···N7^iii^	0.851 (19)	2.150 (19)	2.947 (2)	155.9 (17)

Symmetry codes: (i) −*x*+2, *y*−1/2, −*z*+1/2; (ii) −*x*+1, *y*−1/2, −*z*+1/2; (iii) −*x*+1, *y*+1/2, −*z*+1/2.
